# Changes in *emm* types and superantigen gene content of *Streptococcus pyogenes* causing invasive infections in Portugal

**DOI:** 10.1038/s41598-019-54409-2

**Published:** 2019-12-02

**Authors:** A. Friães, J. Melo-Cristino, M. Ramirez, Portuguese Group for the Study of Streptococcal Infections, Teresa Vaz, Marília Gião, Rui Ferreira, Ana Buschy Fonseca, Henrique Oliveira, Ana Cristina Silva, Hermínia Costa, Margarida Pinto, Odete Chantre, João Marques, Isabel Peres, Isabel Daniel, Ema Canas, Teresa Ferreira, Cristina Marcelo, Lurdes Monteiro, Luís Marques Lito, Teresa Marques, Filomena Martins, Maria Ana Pessanha, Elsa Gonçalves, Teresa Morais, Paulo Lopes, Luísa Felício, Angelina Lameirão, Ana Paula Mota Vieira, Margarida Tomaz, Rosa Bento, Maria Helena Ramos, Ana Paula Castro, Fernando Fonseca, Ana Paula Castro, Graça Ribeiro, Rui Tomé, Celeste Pontes, Luísa Boaventura, Nuno Canhoto, Teresa Afonso, Teresa Pina, Helena Peres, Ilse Fontes, Paulo Martinho, Ana Domingos, Gina Marrão, José Grossinho, Manuela Ribeiro, Alberta Faustino, Adelaide Alves, Maria Paula Pinheiro, R. Semedo, Adriana Coutinho, Luísa Cabral, Olga Neto, Luísa Sancho, José Diogo, Ana Rodrigues, Isabel Nascimento, Elmano Ramalheira, Fernanda Bessa, I. Marques, José Miguel Ribeiro, Maria Antónia Read, Valquíria Alves, Engrácia Raposo, Maria Lurdes Magalhães, Helena Rochas, Anabela Silva, Margarida Rodrigues, Maria Favila Meneses, José Germano de Sousa, Mariana Bettencourt Viana, Isaura Terra, Vitória Rodrigues, Patrícia Pereira, Jesuína Duarte, Paula Pinto, Ezequiel Moreira, João Ataíde Ferreira, Adília Vicente, Paulo Paixão, Natália Novais

**Affiliations:** 1 Instituto de Microbiologia, Instituto de Medicina Molecular, Faculdade de Medicina, Universidade de Lisboa, Lisboa, Portugal; 2 Centro Hospitalar do Barlavento Algarvio, Portimão, Portugal; 3 Hospital de Cascais, Cascais, Portugal; 4 Centro Hospitalar de Coimbra, Coimbra, Portugal; 5 Centro Hospitalar de Entre Douro e Vouga, Santa Maria da Feira, Portugal; 6 Centro Hospitalar de Lisboa Central, Lisboa, Portugal; 7 Centro Hospitalar Lisboa Norte, Lisboa, Portugal; 8 Centro Hospitalar de Lisboa Ocidental, Lisboa, Portugal; 9 Centro Hospitalar de Vila Nova de Gaia/Espinho, Vila Nova de Gaia, Portugal; 10 Centro Hospitalar do Alto Ave, Guimarães, Portugal; 11 Centro Hospitalar do Baixo Alentejo, Beja, Portugal; 12 Centro Hospitalar do Porto, Porto, Portugal; 13 Centro Hospitalar da Póvoa de Varzim/Vila do Conde, Póvoa de Varzim, Portugal; 14 Centro Hospitalar de Trás os Montes e Alto Douro, Vila Real, Portugal; 15 Hospitais da Universidade de Coimbra, Coimbra, Portugal; 16 Centro Hospitalar do Funchal, Funchal, Portugal; 17 Hospital Curry Cabral, Lisboa, Portugal; 18 Hospital de Santa Luzia, Elvas, Portugal; 19 Hospital de Santo André, Leiria, Portugal; 20 Hospital de São João, Porto, Portugal; 21 Hospital de Braga, Braga, Portugal; 22 Hospital Dr. José Maria Grande, Portalegre, Portugal; 23 Hospital do Espírito Santo, Évora, Portugal; 24 Hospital dos SAMS, Lisboa, Portugal; 25 Hospital Prof. Doutor Fernando da Fonseca, Amadora, Portugal; 26 Hospital Garcia de Orta, Almada, Portugal; 27 Hospital Infante D. Pedro, Aveiro, Portugal; 28 Hospital de São Teotónio, Viseu, Portugal; 29 Hospital Pedro Hispano, Matosinhos, Portugal; 30 Instituto Nacional de Saúde Ricardo Jorge, Porto, Portugal; 31 Hospital Reynaldo dos Santos, Vila Franca de Xira, Portugal; 32 Hospital CUF Descobertas, Lisboa, Portugal; 33 Centro Hospitalar do Tâmega e Sousa, Penafiel, Portugal; 34 Hospital Beatriz Ângelo, Loures, Portugal; 35 Centro Hospitalar de Setúbal, Setúbal, Portugal; 36 Hospital Distrital de Santarém, Santarém, Portugal; 37 Centro Hospitalar do Médio Ave, Santo Tirso, Portugal; 38 Hospital de Faro, Faro, Portugal; 39 Centro Hospitalar do Oeste Norte, Caldas da Raínha, Portugal; 40 Hospital da Luz, Lisboa, Portugal; 41 Hospital da Figueira da Foz, Figueira da Foz, Portugal

**Keywords:** Infectious-disease epidemiology, Clinical microbiology, Antimicrobial resistance

## Abstract

Fluctuations in the clonal composition of Group A *Streptococcus* (GAS) have been associated with the emergence of successful lineages and with upsurges of invasive infections (iGAS). This study aimed at identifying changes in the clones causing iGAS in Portugal. Antimicrobial susceptibility testing, *emm* typing and superantigen (SAg) gene profiling were performed for 381 iGAS isolates from 2010–2015. Macrolide resistance decreased to 4%, accompanied by the disappearance of the M phenotype and an increase of the iMLS_B_ phenotype. The dominant *emm* types were: *emm*1 (28%), *emm*89 (11%), *emm*3 (9%), *emm*12 (8%), and *emm*6 (7%). There were no significant changes in the prevalence of individual *emm* types, *emm* clusters, or SAg profiles when comparing to 2006–2009, although an overall increasing trend was recorded during 2000–2015 for *emm*1, *emm*75, and *emm*87. Short-term increases in the prevalence of *emm*3, *emm*6, and *emm*75 may have been driven by concomitant SAg profile changes observed within these *emm* types, or reflect the emergence of novel genomic variants of the same *emm* types carrying different SAgs.

## Introduction

*Streptococcus pyogenes* (Lancefield Group A *Streptococcus*, GAS) can cause a wide spectrum of disease, ranging from superficial infections of the throat and skin, such as pharyngitis and impetigo, to severe invasive infections including necrotizing fasciitis, bacteraemia, and streptococcal toxic shock syndrome. In addition, immune-mediated non-suppurative sequelae, such as acute rheumatic fever and acute glomerulonephritis, remain prevalent in low-income countries and some indigenous populations^1,2^. Despite the global disease burden associated with this pathogen, implicating over 517,000 deaths each year^1^, a safe and effective vaccine has never reached the market^3^. The most promising candidate is a multivalent vaccine based on the hypervariable N-terminal region of the M protein, a major virulence factor and immunogenic protein of GAS. The variability of this N-terminal region is also the basis for the M-serotyping scheme that was used for decades to discriminate GAS strains and that was later replaced by the sequencing of the corresponding hypervariable gene region, known as *emm* typing^4^. More recently, a classification scheme based on the entire sequence of the *emm* gene that clusters closely related M proteins with similar functional and host factor binding properties, was proposed as a typing methodology with potential application for vaccine development^5^.

Penicillin remains the first choice antibiotic treatment for GAS infections, but an association with clindamycin is recommended in severe cases. In addition, both macrolides and lincosamides are important alternatives to β-lactam-allergic patients, although variable macrolide and lincosamide resistance rates can be found among GAS causing infections in different countries^6^.

In Europe and North America, after a decrease throughout most of the 20^th^ century, a resurgence of invasive GAS infections (iGAS) was recorded in the late 1980s^2^. Since then, multiple studies have documented a high incidence of iGAS associated with high morbidity and mortality (https://www.cdc.gov/abcs/reports-findings/surv-reports.html)^7,8^. This was accompanied by a long-term high prevalence of an *emm*1 lineage also known as the virulent M1T1 clone, whose evolutionary pathway has been well documented^9^. However, upsurges of iGAS associated with specific lineages of other *emm* types have also been reported. A few examples are the dissemination of *emm*59 in the US and Canada since the second half of the 2000s decade^10^, the 2008–2009 upsurge of iGAS in the UK due to an *emm*3 lineage with an altered prophage profile^11^, and the spread of an *emm*89 clade lacking the hyaluronic acid capsule synthesis locus in North America and Europe since the 2000s^12–14^. Both the *emm*3 and *emm*89 epidemic lineages were associated with a change in the dominant profile of prophage-encoded genes relative to the previously dominant lineages of the respective *emm* types^11,12^, supporting the usefulness of methodologies like superantigen (SAg) gene profiling as complementary typing methods to further discriminate isolates sharing the same *emm* type^15^.

The molecular surveillance of GAS recovered from human infections worldwide is therefore crucial for providing information on possible shifts in clone prevalence with an impact on vaccine development, as well as for the early detection of clones with enhanced virulence, transmission, or antimicrobial resistance. Previous studies showed that the GAS population causing invasive disease in Portugal is genetically diverse, despite the dominance of the *emm*1 clone^16–18^. From 2000–2005 to 2006–2009, there was a decrease in the diversity of *emm* types, accompanied by a diversification of the SAg gene content of some of the dominant clones^17^. Here we report on the *emm* types, SAg gene profiles, and antimicrobial resistance of 381 iGAS isolates recovered in Portugal during 2010–2015.

## Results

### Demographic data

A total of 381 non-duplicate isolates were received (51 isolates in 2010, 70 in 2011, 62 in 2012, 50 in 2013, 68 in 2014, and 80 in 2015) (dataset available at 10.5281/zenodo.3441765). The great majority of the isolates were recovered from blood (*n* = 330). Other isolate sources included pleural fluid (*n* = 23), ascitic fluid (*n* = 12), synovial fluid (*n* = 10), cerebrospinal fluid (*n* = 4), and bone biopsy (*n* = 2). From the 381 isolates, 193 (51%) were recovered from female patients. Patient age ranged between 1 day and 97 years (median 58 years). The majority of the isolates were recovered from adults (≥18 years, *n* = 295, 77%), mostly from those ≥65 years old (*n* = 151, 40%). Among children, the majority of the isolates were from patients ≤5 years old (*n* = 67, 18%).

### Molecular typing

The 381 iGAS isolates presented a high genetic diversity, comprising 40 different *emm* types, 14 *emm* clusters or singletons, and 52 SAg profiles, all with Simpson’s index of diversity (SID) values > 0.8 (Supplementary Table S1).

**Figure 1:**
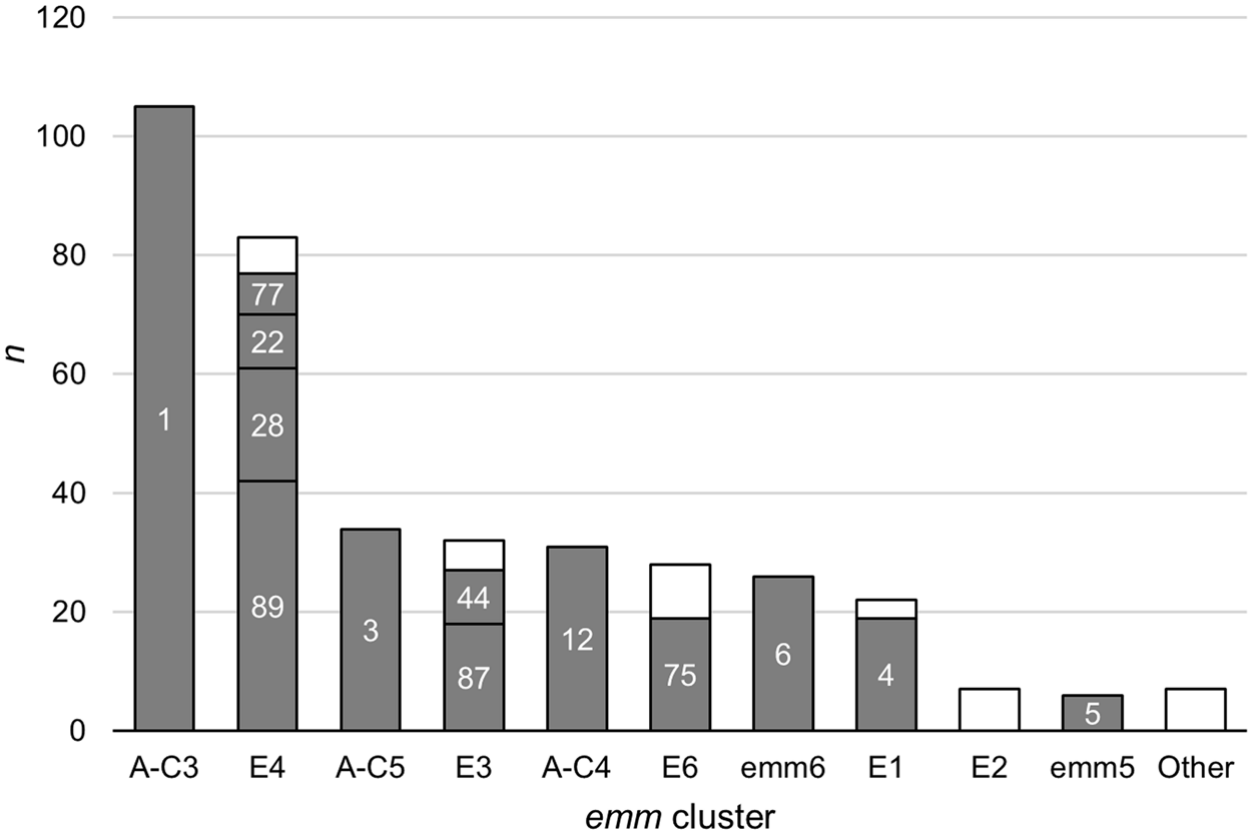
Distribution of GAS isolates recovered from normally sterile sites in Portugal during 2010–2015 according to *emm* cluster and *emm* type. Numbers inside the bars represent the *emm* types included in each cluster. White bars include *emm* types with <5 isolates [E4: *emm*102 (*n* = 2), *emm*2, *emm*73, *emm*84, and *emm*169 (each *n* = 1); E3: *emm*118 (*n* = 2), *emm*9, *emm*82, and *emm*103 (each *n* = 1); E6: *emm*11 (*n* = 4), *emm*81 (*n* = 2), *emm*85, *emm*94, and *emm*99 (each *n* = 1); E1: *emm*78 (*n* = 2) and *emm*165 (*n* = 1); E2: *emm*90, *emm*110 (each *n* = 2), *emm*50, *emm*66, and *emm*104 (each *n* = 1)]. M5 and M6 are singletons belonging to clade Y. “Other” includes *emm* clusters or singletons with <5 isolates each [D4: *emm*70 (*n* = 2), *emm*33, and *emm*43 (each *n* = 1); D2: *emm*71 (*n* = 1); D3: *emm*123 (*n* = 1); M18: *emm*18 (*n* = 1)].

Five *emm* types accounted for 63% of the isolates, namely *emm*1 (28%), *emm*89 (11%), *emm*3 (9%), *emm*12 (8%), and *emm*6 (7%) (Table 1). Although the majority of the *emm* clusters identified in this study were dominated by one *emm* type (Table 1 and Fig. 1), the cluster distribution did not directly reflect the prevalence of the respective dominant *emm* types due to the presence of multiple *emm* types in several clusters, including E3, E4, and E6.

**Table 1:** Properties of 381 GAS isolated from invasive infections in Portugal during 2010–2015.

*emm* cluster (no. of isolates)	*emm* type (no. of isolates)	SAg profile^a^ (no. of isolates)	Antimicrobial resistance^b^ (no. of isolates)
A-C3 (105)	1 (105)	3 (11), 10 (93), 44 (1)	S (104), cMLS_B_ (1)
E4 (83)	2 (1)	48 (1)	S (1)
	22 (9)	21 (1), 40 (1), 53 (4), 91 (1), 95 (1), 103 (1)	S (6), Tet (3)
	28 (19)	10 (2), 15 (1), 24 (9), 27 (6), 54 (1)	S (15), [cMLS_B_, Lev, Bac] (2), [cMLS_B_, Bac] (2)
	73 (1)	16 (1)	Tet (1)
	77 (7)	30 (3), 46 (1), 47 (2), 100 (1)	S (2), [iMLS_B_, Tet] (3), iMLS_B_ (1), Tet (1)
	84 (1)	9 (1)	S (1)
	89 (42)	6 (4), 29 (35), 46 (3)	S (39), cMLS_B_ (2), Lev (1)
	102 (2)	27 (1), 71 (1)	Tet (2)
	169 (1)	40 (1)	Tet (1)
A-C5 (34)	3 (34)	8 (25), 9 (5), 53 (4)	S (34)
E3 (32)	9 (1)	99 (1)	S (1)
	44 (9)	32 (4), 38 (3), 56 (1), 97 (1)	S(9)
	82 (1)	33 (1)	S(1)
	87 (18)	20 (15), 21 (1), 7 (2)	S (18)
	103 (1)	40 (1)	Tet (1)
	118 (2)	29 (1), 44 (1)	Tet (1)
A-C4 (31)	12 (31)	13 (2), 16 (14), 33 (12), 77 (2), 102 (1)	S (30), iMLS_B_ (1)
E6 (28)	11 (4)	16 (1), 29 (2), 46 (1)	S (1), [cMLS_B_, Tet] (2), Tet (1)
	75 (19)	25 (13), 28 (2), 42 (2), 45 (1), 64 (1)	S (19)
	81 (2)	93 (1), 98 (1)	S (2)
	85 (1)	44 (1)	Tet (1)
	94 (1)	35 (1)	S (1)
	99 (1)	46 (1)	Tet (1)
M6 (26)	6 (26)	2 (8), 16 (1), 26 (2), 51 (10), 7 (5)	S (26)
E1 (22)	4 (19)	23 (17), 44 (1), 94 (1)	S (18), Tet (1)
	78 (2)	29 (2)	S (2)
	165 (1)	99 (1)	S (1)
E2 (7)	50 (1)	101 (1)	Tet (1)
	66 (1)	45 (1)	Tet (1)
	90 (2)	95 (2)	Tet (2)
	104 (1)	100 (1)	Tet (1)
	110 (2)	43 (2)	Tet (2)
M5 (6)	5 (6)	29 (6)	S (5), Tet (1)
D4 (4)	33 (1)	67 (1)	Tet (1)
	43 (1)	96 (1)	S (1)
	70 (2)	44 (2)	Tet (2)
D2 (1)	71 (1)	10 (1)	S (1)
D3 (1)	123 (1)	29 (1)	S (1)
M18 (1)	18 (1)	4 (1)	S (1)

^a^The SAg genes present in each profile are indicated in Supplementary Table S2.

^b^S, susceptibility to all antimicrobials tested; cMLS_B_, presenting the cMLS_B_ phenotype of macrolide resistance; iMLS_B_, presenting the iMLS_B _phenotype of macrolide resistance; Tet, resistance to tetracycline; Lev, nonsusceptibility to levofloxacin; Bac, resistance to bacitracin.

Among the studied isolates, *emm*1 (and, as such, cluster A-C3) and *emm* cluster E4 were slightly overrepresented among paediatric and adult patients, respectively (*p = *0.013 and *p* = 0.025, respectively), and *emm* cluster E1 was more prevalent in males (*p* = 0.008). However, all these associations lost statistical significance after the false-discovery rate (FDR) correction.

In line with the results obtained in previous studies^17,18^, the chromosomal SAg genes *speG* and *smeZ* were detected in the great majority of isolates (*n* = 354 and 376, respectively), followed by *speC* (*n* = 190), *speA* (*n* = 167), *speJ* (*n* = 160), *speK* (*n* = 91), *ssa* (*n* = 89), *speH* (*n* = 64), *speI* (*n* = 58), *speM* (*n* = 33), and *speL* (*n* = 32) (Supplementary Table S2). With the exception of *emm*5, all *emm* types with >5 isolates included multiple SAg profiles (Table 1).

The absence of the *hasABC* locus encoding the GAS capsule biosynthesis pathway was used as a surrogate for the identification of the recently emerged acapsular *emm*89 clade^12^. Among the 42 iGAS isolates presenting *emm*89 in this study, only 3 (isolated in 2010 and 2011) were positive for the capsule locus.

### Antimicrobial resistance

All 381 isolates were susceptible to penicillin, chloramphenicol, vancomycin, and linezolid. Fourteen isolates (4%) were resistant to erythromycin (Table 1), of which nine were constitutively resistant to clindamycin (cMLS_B_ phenotype) and carried the *erm*(B) gene, while five presented inducible resistance to clindamycin (iMLS_B_ phenotype), harbouring the *erm*(TR) gene. Despite the small number of macrolide resistant isolates, their genetic diversity was high [SID (CI_95%_) = 0.846 (0.755–0.937)], with six different *emm* types identified.

Tetracycline resistance was detected in 30 isolates (8%) (Table 1), of which 23 carried the *tet*(M) gene, 3 carried both *tet*(L) and *tet*(M), 3 harboured *tet*(O), and 1 presented *tet*(L) only (dataset available at 10.5281/zenodo.3441765). Tetracycline-resistant isolates were also very diverse [19 different *emm* types, SID (CI_95%_) = 0.963 (0.937–0.989)]. Five isolates were also resistant to erythromycin, including two *emm*11 isolates carrying *erm*(B) and *tet*(M), and three *emm*77 isolates carrying *erm*(TR) and *tet*(O).

In agreement with previously studied periods^17,18^, during 2010–2015 bacitracin resistance remained restricted to an *emm*28 lineage expressing the cMLS_B_ phenotype of macrolide resistance (*n* = 4, Table 1).

Two isolates presented intermediate resistance to levofloxacin (MIC = 4 and 6 µg/ml). Both belonged to *emm*28, were resistant to erythromycin, clindamycin (cMLS_B_), and bacitracin, and carried the mutation S79Y in the quinolone resistance determining regions (QRDR) of the *parC* gene. One *emm*89 isolate presented high-level levofloxacin resistance (MIC > 32 µg/ml) and carried mutation S79F in *parC* and mutation E85K in *gyrA*.

## Discussion

The iGAS isolates recovered throughout Portugal between 2010 and 2015 were genetically diverse, with SID values similar to the ones obtained for iGAS isolates recovered during 2006–2009^17^. Still, the five most prevalent *emm* types, namely *emm* types 1, 89, 3, 12, and 6, comprised 63% of the isolates, with *emm*1 persisting as the leading invasive *emm* type (28%). Twenty-one of the forty *emm* types identified in this study (94% of the isolates) are included in the 30-valent M protein-based vaccine currently under development. This vaccine could potentially cover up to 96% of the isolates of this study, considering the presumed cross-protection against a number of non-vaccine serotypes^19^. These results are in agreement with the overall scenario in Europe and the US in contemporary periods, although with some variations in the ranking of the top *emm* types^7,8,20–26^. In contrast, remarkable heterogeneity is found in the Southern hemisphere and developing regions, where the diversity of *emm* types is significantly higher, resulting in a much lower estimated coverage of the 30-valent vaccine^27–29^.

**Figure 2:**
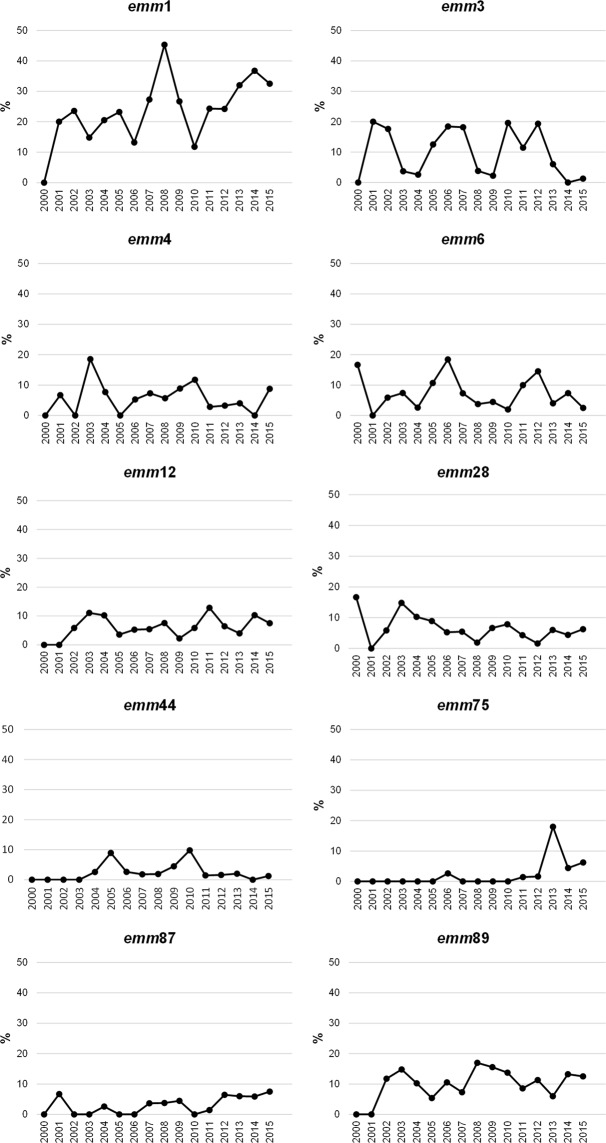
Yearly prevalence (%) of *emm* types with ≥20 isolates in 2000–2015. Isolates from 2000–2009 were characterised previously^16,17^.

Since we have been following the molecular epidemiology of iGAS in Portugal from 2000 onwards^16–18^, the yearly distribution of *emm* types with ≥20 isolates in 2000–2015 was analysed over the 16 studied years (Fig. 2). For most *emm* types there were yearly fluctuations without any specific trend. However, an increasing trend was observed for *emm*1 (*p* = 0.008), *emm*75 (*p* < 0.001), and *emm*87 (*p* = 0.007), which remained significant after FDR correction. The increase in *emm*1 further reinforces the prolonged success of the contemporary *emm*1 clone in causing iGAS in temperate climate regions. In Portugal, this clone has been dominant among iGAS for at least 15 years and was shown to be overrepresented in isolates recovered from normally sterile sites compared to isolates from pharyngeal and skin and soft tissue infections^17,18,30^. In line with our results, in recent years, the prevalence of *emm*1 among iGAS in Europe and the US varied between 22% and 32%^7,20–25^; the exceptions being the lower prevalence in Finland (12%)^8^ and higher in Scotland, where this *emm* type accounted for 66% of iGAS in 2011–2015^26^.

The increasing trend in *emm*75 is in agreement with the rise in prevalence of this *emm* type from 0.5% in 2006–2009 to 5% in 2010–2015 (*p* = 0.006), which was not significant after FDR correction. This increase in *emm*75 was somewhat surprising, since we previously found this *emm* type to be significantly underrepresented among iGAS when compared with pharyngeal isolates recovered in the same period in Portugal^18^. In agreement, an *emm*75 strain was recently selected for a controlled human infection model of GAS pharyngitis based on its limited virulence^31^. In our previous study including iGAS and pharyngitis isolates, a high diversity among *emm*75 isolates was observed^18^. Among the *emm*75 isolates from 2010–2015, five different SAg profiles were identified, but 13/19 isolates presented SAg25. Previously, only two *emm*75-SAg25 isolates had been identified in Portugal, both recovered from pharyngeal infections in the period of 2000–2005^18^. The increasing trend in *emm*75 among iGAS could result from the emergence of this particular lineage from 2013 onwards. At present, it is not possible to know if this lineage is particularly prone to causing invasive disease or if it increased equally among non-invasive infections in Portugal.

The prevalence of *emm*87 has been gradually increasing among iGAS in Portugal, with no apparent new lineage emerging in recent years when considering SAg profiles, which remained the same (mostly SAg20). Isolates of *emm*87 have been associated with familial and hospital clusters of iGAS and proposed to be highly transmissible^32,33^, but have not been specifically associated with iGAS when compared with contemporary non-invasive isolates^18,34^.

**Figure 3:**
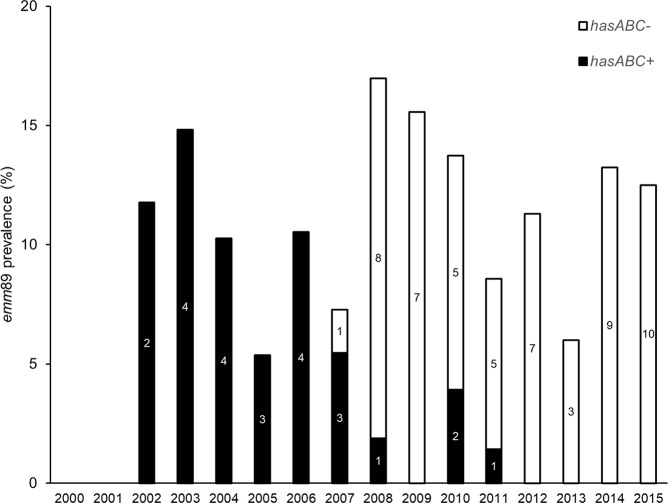
Yearly distribution of invasive *emm*89 isolates with (filled bars) and without (open bars) the *hasABC* locus. Numbers inside the bars represent number of isolates. Data from 2000–2009 was previously published^12^.

In Portugal, the recent acapsular *emm*89 clade emerged among iGAS in 2007 and quickly outcompeted the previously circulating *emm*89 isolates carrying the *hasABC* locus^12^. Accordingly, among the 42 *emm*89 isolates recovered between 2010 and 2015, only 3 isolates carried the capsule locus (Fig. 3). The prevalence of *emm*89 among iGAS did not present an increasing trend, nor did it increase significantly in the period following the introduction of the new clade when compared with previous years, in contrast to what we reported among isolates from skin and soft tissue infections^30^. This indicates that the new clade was highly successful in outcompeting the previously circulating *emm*89 isolates in all infection types, but is not associated with an enhanced ability to cause infection in normally sterile sites.

Between 2000–2005 and 2006–2009 a diversification of SAg profiles within *emm* types 1, 28 and 44 was noted, as well as a shift in the dominant SAg profile among *emm*89 isolates that was correlated with the emergence of the acapsular clade^12,17^. The comparison of the SID of the SAg profiles identified within *emm* types with ≥5 isolates in each of the two most recent study periods (2006–2009 and 2010–2015) showed a significant diversification of SAg profiles for *emm*3 and *emm*6 (*p* = 0.009 and *p* < 0.001, respectively) (Table 2). SAg8 was dominant among *emm*3 isolates in all studied periods, but up to 2009 only one isolate presented a different SAg profile (SAg53), while in 2010–2015 there were four isolates with SAg53 and five isolates with SAg9. Regarding *emm*6, up to 2008 all isolates presented SAg2. In 2009 SAg51 emerged and became the most common SAg profile in 2010–2015 (*n* = 9), followed by SAg2 (*n* = 8) and three other SAg profiles that emerged in this period, namely SAg72 (*n* = 5), SAg26 (*n* = 2), and SAg16 (*n* = 1). Further studies are needed to clarify if the isolates presenting the new SAg profiles within *emm*3 and *emm*6 emerged from the previously dominant *emm*3-SAg8 and *emm*6-SAg2 lineages by loss or gain of SAg genes, or if they represent distinct genetic clades that could underlie the rise in prevalence of both *emm* types during 2010–2012 (Fig. 2).

**Table 2:** Simpson’s index of diversity (SID) and 95% confidence intervals (CI_95%_) of the SAg profiles within *emm* types with ≥5 isolates in 2006–2009 and in 2010–2015.

*emm*	2006–2009^a^			2010–2015			*p*
	*n*	No. partitions	SID SAg profile (95% CI)	*n*	No. partitions	SID SAg profile (95% CI)	
1	56	4	0.338 (0.191–0.486)	105	3	0.206 (0.108–0.304)	0.135
3	20	2	0.100 (0.000–0.275)	34	3	0.437 (0.255–0.619)	**0.009**
4	13	3	0.295 (0.000–0.603)	19	3	0.205 (0.000–0.442)	0.766
5	6	2	0.333 (0.000–0.739)	6	1	0.000 (0.000–0.000)	NA
6	15	2	0.133 (0.000–0.357)	26	5	0.742 (0.658–0.825)	**<0.001**
12	10	2	0.533 (0.409–0.657)	31	5	0.658 (0.560–0.757)	0.113
28	9	4	0.750 (0.579–0.921)	19	5	0.696 (0.556–0.836)	0.625
44	5	4	0.900 (0.725–1.000)	9	4	0.750 (0.579–0.921)	0.305
87	6	2	0.333 (0.000–0.739)	18	3	0.307 (0.047–0.568)	0.784
89	24	3	0.518 (0.329–0.708)	42	3	0.298 (0.126–0.471)	0.086

^a^Isolates from 2006–2009 were characterised previously^17^.

**Figure 4:**
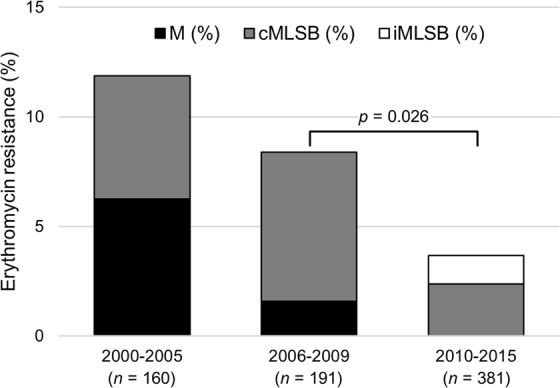
Prevalence of erythromycin resistance and of macrolide resistance phenotypes among isolates recovered from invasive infections in Portugal during 2000–2015. The numbers below each period represent the total number of iGAS isolates recovered. Data from 2000–2005 and 2006–2009 was previously published^17,18^.

Erythromycin resistance (4%) decreased relative to the previously studied period of 2006–2009^17^ (8%, *p* = 0.026) (Fig. 4). The overall decreasing trend in macrolide resistance recorded among invasive GAS in the period of 2000–2015 (*p* < 0.001) mirrors the one previously reported for isolates recovered from pharyngitis and skin and soft tissue infections^30,35^. Despite this decrease, the genetic diversity of the macrolide resistant isolates remained high.

The genetic determinants of tetracycline resistance are often horizontally transferred together with macrolide resistance determinants in the same mobile genetic elements^36^. Among the 30 resistant iGAS isolates from 2010–2015 in Portugal (8%), only 5 were also resistant to erythromycin, 3 of which belong to a lineage of *emm*77-SAg30 isolates carrying *erm*(TR) and *tet*(O) that had not been previously identified among iGAS in Portugal. Although the tetracycline resistance rate did not decrease significantly relative to 2006–2009, an overall decreasing trend was observed during 2000–2015 (*p* = 0.002).

A limitation of this study is that isolate submission was voluntary, without any audit, preventing us from controlling any possible bias on the selection of the isolates submitted by each lab. Although we expect that not all isolates recovered from iGAS were submitted, the inclusion of 40 laboratories distributed throughout the country provided us with a representative collection of isolates, limiting the impact that any strain selection bias could have on the results and conclusions of the study. Screening of SAg and resistance genes by PCR alone presents another limitation given the possible occurrence of false-positives and false-negatives. In order to reduce the potential impact of this limitation on the results, we have used carefully optimised multiplex PCR reaction conditions, including both positive and negative controls in each reaction^15,17^. The high correlation between SAg profiles and the results of other typing methods^15^, as well as between the resistance genotypes determined and the respective resistance phenotypes and lineages, supports the accuracy of the PCR results.

This is the first study providing detailed molecular epidemiological data on iGAS infections in a Southern European country in the current decade. The results suggest that the *emm* type and *emm* cluster composition of GAS causing invasive disease in Portugal has remained stable since the second half of the 2000s decade, presenting no major changes in prevalence of individual *emm* types or clusters^17^. However, there have been changes in the SAg gene content within multiple *emm* types, which may reflect the ongoing horizontal transfer of phage-encoded genes between GAS lineages, or the emergence of new genetic clades. In some cases, these changes seem to be associated with temporal fluctuations in the prevalence of the respective *emm* types. Streptococcal SAgs can directly contribute to the emergence of new successful lineages through their role in virulence and the immune response^37^. On the other hand, changes in SAg gene content reflect the loss and acquisition of prophages that often carry other virulence factors or antimicrobial resistance determinants that could also contribute to the success of those lineages^38^. Given that the emergence of clades with increased success within previously circulating *emm* types has been reported in multiple occasions^11,13^, the continued molecular surveillance of GAS infections using methods capable of further discriminating isolates sharing the same *emm* type is critical for the identification of the emergence of novel lineages which could drive increases in iGAS disease.

## Materials and Methods

### Bacterial isolates

Forty clinical microbiology laboratories distributed throughout Portugal were asked to submit, on a voluntary basis, all GAS isolated from normally sterile sites between January 2010 and December 2015. The study was approved by the Institutional Review Board of the Centro Académico de Medicina de Lisboa. Since only anonymized demographic patient information was used and the samples used were collected within the normal diagnostic procedure by the attending physician, the study was exempt from obtaining written informed consent from the patients. All methods were performed in accordance with the relevant guidelines and regulations. Strains were identified by the submitting laboratories and confirmed in our laboratory by colony morphology, β-haemolysis, and the presence of the characteristic Lancefield group A antigen (Oxoid, Basingstoke, UK).

### Molecular typing

The *emm* type was determined for all isolates according to the protocols and recommendations of the CDC (http://www.cdc.gov/streplab/groupa-strep/emm-typing-protocol.html), and the first 240 bases of each sequence were compared to the sequences deposited in the CDC *emm* database using the CDC BLAST tool (http://www2a.cdc.gov/ncidod/biotech/strepblast.asp). The presence of 11 SAg genes (*speA*, *speC*, *speG*, *speH*, *speI*, *speJ*, *speK*, *speL*, *speM*, *smeZ*, and *ssa*) was tested by two previously described multiplex PCR reactions, using the chromosomally encoded genes *speB* and *speF* as positive control fragments^15^. All *emm*89 isolates were screened for the presence of the *has* locus by PCR^12^.

### Antimicrobial susceptibility testing

Susceptibility tests were performed for all isolates by disk diffusion according to the guidelines and interpretative criteria of the Clinical and Laboratory Standards Institute (CLSI)^39^, using the following disks (Oxoid, Basingstoke, UK): penicillin, vancomycin, erythromycin, tetracycline, levofloxacin, chloramphenicol, clindamycin, and linezolid. Macrolide resistance phenotypes were determined by the double-disk test^39^. *E*-test strips (BioMérieux, Marcy l’Etoile, France) and CLSI interpretative criteria^39^ were used for MIC determination in levofloxacin non-susceptible isolates and in all cases of intermediate susceptibility by disk diffusion. Susceptibility to bacitracin was determined using BD BBL^TM^ Taxo^TM^ A Disks (Becton, Dickinson and Company, Sparks, MD, USA).

### Detection of genetic determinants of antimicrobial resistance

The screening for the genetic determinants of resistance to macrolides, tetracycline and fluoroquinolones was performed as previously described^17^. Briefly, erythromycin-resistant isolates were tested for the presence of the *mef*, *erm*(A), and *erm*(B) genes by multiplex PCR, followed by a second PCR to distinguish between *mef*(A) and *mef*(E) in *mef*-positive isolates. Tetracycline-resistant isolates were PCR-screened for the presence of the *tet*(K), *tet*(L), *tet*(M), and *tet*(O) genes. For levofloxacin non-susceptible isolates, the QRDRs of the *gyrA* and *parC* genes were amplified by PCR and sequenced.

### Statistical analysis

The diversity of the isolates according to different typing methods was evaluated using the SID with corresponding 95% confidence intervals (CI_95%_)^40^, calculated using an online tool (http://www.comparingpartitions.info). Two-tailed Fisher’s exact test and odds ratios were used to identify significant pairwise associations. The Cochran-Armitage test was used to evaluate trends. The *p*-values for multiple tests were corrected using the FDR linear procedure^41^. Values of *p* < 0.05 were considered statistically significant.

## Supplementary information

Supplementary Information

## Data Availability

The datasets generated and analysed during the current study are available in the Zenodo repository, 10.5281/zenodo.3441765.

## References

[1] Carapetis JR, Steer AC, Mulholland EK, Weber M (2005). The global burden of group A streptococcal diseases. Lancet Infect. Dis..

[2] Walker MJ (2014). Disease manifestations and pathogenic mechanisms of group A *Streptococcus*. Clin. Microbiol. Rev..

[3] Fischetti, V. A. Vaccine approaches to protect against group A streptococcal pharyngitis. *Microbiol Spectr* **7** (2019). 10.1128/microbiolspec.gpp3-0010-2018 PMC11026073 31111819

[4] Beall B, Facklam R, Thompson T (1996). Sequencing *emm*-specific PCR products for routine and accurate typing of group A streptococci. J. Clin. Microbiol..

[5] Sanderson-Smith M (2014). A systematic and functional classification of *Streptococcus pyogenes* that serves as a new tool for molecular typing and vaccine development. J Infect Dis.

[6] Silva-Costa C, Friães A, Ramirez M, Melo-Cristino J (2015). Macrolide-resistant *Streptococcus pyogenes*: prevalence and treatment strategies. Expert Rev Anti Infect Ther.

[7] Naseer U, Steinbakk M, Blystad H, Caugant DA (2016). Epidemiology of invasive group A streptococcal infections in Norway 2010–2014: A retrospective cohort study. Eur J Clin Microbiol Infect Dis.

[8] Smit PW (2015). Epidemiology and *emm* types of invasive group A streptococcal infections in Finland, 2008–2013. Eur J Clin Microbiol Infect Dis.

[9] Nasser W (2014). Evolutionary pathway to increased virulence and epidemic group A *Streptococcus* disease derived from 3,615 genome sequences. Proc. Natl. Acad. Sci. USA.

[10] Fittipaldi N (2012). Full-genome dissection of an epidemic of severe invasive disease caused by a hypervirulent, recently emerged clone of group A. Streptococcus. Am. J. Pathol..

[11] Afshar B (2017). Enhanced nasopharyngeal infection and shedding associated with an epidemic lineage of *emm*3 group A *Streptococcus*. Virulence.

[12] Friães A (2015). Emergence of the same successful clade among distinct populations of *emm*89 *Streptococcus pyogenes* in multiple geographic regions. mBio.

[13] Turner CE (2015). Emergence of a new highly successful acapsular group A *Streptococcus* clade of genotype *emm*89 in the United Kingdom. mBio.

[14] Zhu L (2015). A molecular trigger for intercontinental epidemics of group A *Streptococcus*. Journal of Clinical Investigation.

[15] Friães A, Pinto FR, Silva-Costa C, Ramirez M, Melo-Cristino J (2013). Superantigen gene complement of *Streptococcus pyogenes*-relationship with other typing methods and short-term stability. Eur. J. Clin. Microbiol. Infect. Dis..

[16] Friães A, Ramirez M, Melo-Cristino J (2007). & the Portuguese Group for the Study of Streptococcal Infections. Nonoutbreak surveillance of group A streptococci causing invasive disease in Portugal identified internationally disseminated clones among members of a genetically heterogeneous population. J. Clin. Microbiol..

[17] Friães A, Lopes JP, Melo-Cristino J, Ramirez M (2013). & Portuguese Group for the Study of Streptococcal Infections. Changes in *Streptococcus pyogenes* causing invasive disease in Portugal: Evidence for superantigen gene loss and acquisition. Int. J. Med. Microbiol..

[18] Friães A, Pinto FR, Silva-Costa C, Ramirez M, Melo-Cristino J (2012). Group A streptococci clones associated with invasive infections and pharyngitis in Portugal present differences in *emm* types, superantigen gene content and antimicrobial resistance. BMC Microbiol..

[19] Dale JB, Penfound TA, Chiang EY, Walton WJ (2011). New 30-valent M protein-based vaccine evokes cross-opsonic antibodies against non-vaccine serotypes of group A streptococci. Vaccine.

[20] Meehan M, Murchan S, Gavin PJ, Drew RJ, Cunney R (2018). Epidemiology of an upsurge of invasive group A streptococcal infections in Ireland, 2012-2015. J. Infect..

[21] Imöhl M, Fitzner C, Perniciaro S, van der Linden M (2017). Epidemiology and distribution of 10 superantigens among invasive *Streptococcus pyogenes* disease in Germany from 2009 to 2014. PLoS One.

[22] Olafsdottir LB (2014). Invasive infections due to *Streptococcus pyogenes*: seasonal variation of severity and clinical characteristics, Iceland, 1975 to 2012. Euro Surveill..

[23] Lambertsen LM, Ingels H, Schønheyder HC, Hoffmann S (2014). & Danish Streptococcal Surveillance Collaboration Group 2011. Nationwide laboratory-based surveillance of invasive beta-haemolytic streptococci in Denmark from 2005 to 2011. Clin. Microbiol. Infect..

[24] Nelson GE (2016). Epidemiology of invasive group A streptococcal infections in the United States, 2005-2012. Clin. Infect. Dis..

[25] Chochua S (2017). Population and whole genome sequence based characterization of invasive group A streptococci recovered in the United States during 2015. mBio.

[26] Lindsay DSJ (2016). Circulating *emm* types of *Streptococcus pyogenes* in Scotland: 2011–2015. Journal of Medical Microbiology.

[27] Baroux N (2014). The *emm*-cluster typing system for group A *Streptococcus* identifies epidemiologic similarities across the Pacific region. Clin Infect Dis..

[28] Williamson DA (2016). Comparative M-protein analysis of *Streptococcus pyogenes* from pharyngitis and skin infections in New Zealand: Implications for vaccine development. BMC Infectious Diseases.

[29] Abraham T, Sistla S (2019). Decoding the molecular epidemiology of group A *Streptococcus* - an Indian perspective. J. Med. Microbiol..

[30] Pato C, Melo-Cristino J, Ramirez M, Friães A (2018). & Portuguese Group for the Study of Streptococcal Infections. *Streptococcus pyogenes* causing skin and soft tissue infections are enriched in the recently emerged *emm*89 clade 3 and are not associated with abrogation of CovRS. Front Microbiol.

[31] Osowicki, J. *et al*. A controlled human infection model of group A *Streptococcus* pharyngitis: which strain and why? *mSphere* **4** (2019). 10.1128/mSphere.00647-18 PMC6374595 30760615

[32] Flores AR, Luna RA, Runge JK, Shelburne SA, Baker CJ (2017). Cluster of fatal group A streptococcal *emm*87 infections in a single family: molecular basis for invasion and transmission. J. Infect. Dis..

[33] Montes M, Tamayo E, Oñate E, Pérez-Yarza EG, Pérez-Trallero E (2013). Outbreak of *Streptococcus pyogenes* infection in healthcare workers in a paediatric intensive care unit: transmission from a single patient. Epidemiol. Infect..

[34] Ekelund K (2005). Variations in *emm* type among group A streptococcal isolates causing invasive or noninvasive infections in a nationwide study. J. Clin. Microbiol..

[35] Silva-Costa C, Ramirez M, Melo-Cristino J (2015). & Portuguese Group for Study of Streptococcal Infections. Declining macrolide resistance in *Streptococcus pyogenes* in Portugal (2007-13) was accompanied by continuous clonal changes. J. Antimicrob. Chemother..

[36] Varaldo PE, Montanari MP, Giovanetti E (2009). Genetic elements responsible for erythromycin resistance in streptococci. Antimicrob. Agents Chemother..

[37] Shannon, B. A., McCormick, J. K. & Schlievert, P. M. Toxins and superantigens of group A streptococci. *Microbiol Spectr* **7** (2019). 10.1128/microbiolspec.gpp3-0054-2018 PMC11590448 30737912

[38] McShan, W. M., McCullor, K. A. & Nguyen, S. V. The bacteriophages of *Streptococcus pyogenes*. *Microbiol Spectr* **7** (2019). 10.1128/microbiolspec.gpp3-0059-2018 PMC11314938 31111820

[39] Clinical and Laboratory Standards Institute. *Performance standards for antimicrobial susceptibility testing*. *CLSI document M100*. (Clinical and Laboratory Standards Institute, Pennsylvania, USA, 2018).

[40] Carriço JA (2006). Illustration of a common framework for relating multiple typing methods by application to macrolide-resistant *Streptococcus pyogenes*. J. Clin. Microbiol..

[41] Benjamini Y, Hochberg Y (1995). Controlling the False Discovery Rate: a practical and powerful approach to multiple testing. J. R. Stat. Soc..

