# Supplementary material for: Changes in emm types and superantigen gene content of Streptococcus pyogenes causing invasive infections in Portugal
Source: Sci Rep. 2019 Dec 2;9:18051. doi: 10.1038/s41598-019-54409-2 (PMC6888849; doi:10.1038/s41598-019-54409-2)
Supplement: Supplementary file 1 — Supplementary Information [file 41598_2019_54409_MOESM1_ESM.pdf]

Supplementary Information

Changes in *emm* types and superantigen gene content of *Streptococcus pyogenes* causing invasive infections in Portugal

A. Friães[1,*], J. Melo-Cristino[1], M. Ramirez[1], the Portuguese Group for the Study of Streptococcal Infections

[1]Instituto de Microbiologia, Instituto de Medicina Molecular, Faculdade de Medicina, Universidade de Lisboa, Lisboa, Portugal

* Correspondence:
Ana Friães
Faculdade de Medicina de Lisboa
Av. Professor Egas Moniz,
Ed. Egas Moniz, P2A
1649-028 Lisboa
Portugal
afriaes@fm.ul.pt
+351 217999458

Supplementary Table S1 – Simpson's index of diversity (SID) and respective 95% confidence intervals ($CI_{95\%}$) for *emm* cluster, *emm* type, and superantigen (SAg) profile among 381 GAS isolated from invasive infections in Portugal during 2010-2015.

	No. partitions	SID	$CI_{95\%}$
***emm* cluster**	14	0.843	0.824-0.862
***emm* type**	40	0.883	0.862-0.927
**SAg profile**	52	0.906	0.886-0.926

Supplementary Table S2 – SAg profiles identified among 381 GAS isolated from invasive infections in Portugal during 2010-2015.

**SAg profile[a] (*n*)**	***speA***	***speC***	***speG***	***speH***	***speI***	***speJ***	***speK***	***speL***	***speM***	***ssa***	***smeZ***
2 (8)	+	+	+	-	-	-	+	-	-	-	+
3 (11)	+	+	+	-	-	+	-	-	-	-	+
4 (1)	+	+	+	-	-	-	-	+	+	-	+
8 (25)	+	-	+	-	-	-	+	-	-	+	+
9 (6)	+	-	+	-	-	-	+	-	-	-	+
10 (96)	+	-	+	-	-	+	-	-	-	-	+
13 (2)	-	+	+	+	+	-	-	-	-	+	+
15 (1)	-	+	+	+	+	+	+	-	-	-	+
16 (17)	-	+	+	+	+	-	-	-	-	-	+
20 (15)	-	+	+	-	-	+	-	-	-	+	+
21 (2)	-	+	+	-	-	-	-	-	-	+	+
23 (17)	-	+	-	-	-	-	-	-	-	+	+
24 (9)	-	+	+	-	-	+	+	-	-	-	+
25 (13)	-	+	+	-	-	-	+	+	+	-	+
26 (6)	-	+	+	-	-	-	+	-	-	-	+
27 (9)	-	+	+	-	-	+	-	-	-	-	+
28 (2)	-	+	+	-	-	-	-	+	+	-	+
29 (47)	-	+	+	-	-	-	-	-	-	-	+
30 (3)	-	+	-	-	-	-	-	-	-	-	+
32 (4)	-	-	+	+	+	+	-	-	-	+	+
33 (13)	-	-	+	+	+	-	-	-	-	-	+
35 (1)	-	-	+	+	-	-	-	-	-	-	+
38 (3)	-	-	+	-	-	+	-	-	-	+	+
40 (3)	-	-	+	-	-	-	-	-	-	+	+
42 (2)	-	-	+	-	-	-	+	+	+	-	+
43 (2)	-	-	+	-	-	-	+	-	-	-	+
44 (6)	-	-	+	-	-	+	-	-	-	-	+
45 (2)	-	-	+	-	-	-	-	+	+	-	+
46 (6)	-	-	+	-	-	-	-	-	-	-	+
47 (2)	-	-	-	-	-	-	-	-	-	-	+
48 (1)	-	-	+	-	-	-	-	+	+	-	-
51 (10)	+	+	+	+	+	-	+	-	-	-	+
53 (8)	+	-	+	-	-	-	-	-	-	+	+
54 (1)	-	+	+	+	+	+	-	-	-	-	+
56 (1)	-	-	+	+	-	+	-	-	-	-	-
64 (1)	-	+	+	+	+	-	-	+	+	-	+
67 (1)	-	-	+	+	-	+	-	-	-	+	+
71 (1)	-	+	+	-	-	+	-	+	+	-	+
72 (5)	-	+	+	+	+	-	+	-	-	-	+
77 (2)	-	-	+	+	+	-	-	-	-	+	+
91 (1)	+	+	+	-	-	-	-	-	-	+	+
93 (1)	-	+	+	+	-	-	-	-	-	-	-
94 (1)	-	+	-	-	-	-	+	-	-	+	+
95 (3)	-	+	+	-	-	-	-	+	+	+	+

Supplementary Table S2 ***(continued)***

**SAg profile[a] (*n*)**	***speA***	***speC***	***speG***	***speH***	***speI***	***speJ***	***speK***	***speL***	***speM***	***ssa***	***smeZ***
96 (1)	-	+	+	-	-	+	+	+	+	-	+
97 (1)	-	-	+	+	-	+	-	+	+	-	-
98 (1)	-	-	+	+	-	-	-	+	+	-	-
99 (2)	-	-	-	-	-	-	+	-	-	-	+
100 (2)	-	-	-	-	-	-	-	+	+	-	+
101 (1)	-	-	+	-	-	-	-	-	+	-	+
102 (1)	+	+	+	+	+	-	-	-	-	+	+
103 (1)	-	+	+	+	+	-	-	+	+	+	+

[a] The numbering of the SAg profiles follows the one adopted previously (Friães *et al.* Eur J Clin Microbiol Infect Dis 2013, 32:115-25; Friães *et al.* Int J Med Microbiol 2013, 303:505-513; Silva-Costa *et al.* Pediatr Infect Dis J 2014, 33:306-10; Pato *et al.* Front Microbiol 2018, 9).
